# Outlier detection in BLAST hits

**DOI:** 10.1186/s13015-018-0126-3

**Published:** 2018-03-22

**Authors:** Nidhi Shah, Stephen F. Altschul, Mihai Pop

**Affiliations:** 1 Department of Computer Science and Center for Bioinformatics and Computational Biology, University of Maryland, College Park, 20742 USA; 2 Computational Biology Branch, NCBI, NLM, NIH, Bethesda, 20894 USA

**Keywords:** Taxonomy classification, Metagenomics, Sequence alignment, Outlier detection

## Abstract

**Background:**

An important task in a metagenomic analysis is the assignment of taxonomic labels to sequences in a sample. Most widely used methods for taxonomy assignment compare a sequence in the sample to a database of known sequences. Many approaches use the best BLAST hit(s) to assign the taxonomic label. However, it is known that the best BLAST hit may not always correspond to the best taxonomic match. An alternative approach involves phylogenetic methods, which take into account alignments and a model of evolution in order to more accurately define the taxonomic origin of sequences. Similarity-search based methods typically run faster than phylogenetic methods and work well when the organisms in the sample are well represented in the database. In contrast, phylogenetic methods have the capability to identify new organisms in a sample but are computationally quite expensive.

**Results:**

We propose a two-step approach for metagenomic taxon identification; i.e., use a rapid method that accurately classifies sequences using a reference database (this is a filtering step) and then use a more complex phylogenetic method for the sequences that were unclassified in the previous step. In this work, we explore whether and when using top BLAST hit(s) yields a correct taxonomic label. We develop a method to detect outliers among BLAST hits in order to separate the phylogenetically most closely related matches from matches to sequences from more distantly related organisms. We used modified BILD (Bayesian Integral Log-Odds) scores, a multiple-alignment scoring function, to define the outliers within a subset of top BLAST hits and assign taxonomic labels. We compared the accuracy of our method to the RDP classifier and show that our method yields fewer misclassifications while properly classifying organisms that are not present in the database. Finally, we evaluated the use of our method as a pre-processing step before more expensive phylogenetic analyses (in our case TIPP) in the context of real 16S rRNA datasets.

**Conclusion:**

Our experiments make a good case for using a two-step approach for accurate taxonomic assignment. We show that our method can be used as a filtering step before using phylogenetic methods and provides a way to interpret BLAST results using more information than provided by E-values and bit-scores alone.

## Background

One of the goals of metagenomic analyses is to characterize the biological diversity of microbial communities. This is usually achieved by targeted amplicon sequencing of the 16S rRNA gene, either as a whole gene or focused on a hypervariable region within the gene [1]. The 16S rRNA gene is commonly used for this purpose because it is universally found in bacteria and contains a combination of highly conserved and highly variable regions. Advances in sequencing technology, targeted to a specific gene, have generated millions to hundreds of millions of reads per study [2]. Assigning accurate taxonomic labels to these reads is one of the critical steps for downstream analyses.

The most common approach for assigning taxonomic labels to reads involves comparing them to a database of sequences from known organisms. These similarity-based methods typically run rapidly and work well when organisms in the sample are well represented in the database. However, a majority of microorganisms cannot be easily cultured in laboratories, and even if they are culturable, a smaller number have been sequenced. Thus, not all environmental organisms may be represented in the sequence database. This prevents the similarity-based methods from accurately characterizing organisms within a sample that are only distantly related to the sequences in the reference database. Phylogenetic-tree based methods can characterize novel organisms within a sample by statistically modeling the evolutionary processes that generated these sequences [3, 4]. However, such methods incur a high computational cost, limiting their applicability in the context of the large datasets generated in contemporary studies. Ideally, we would want to use similarity-based methods to assign labels to sequences from known organisms, and to use phylogenetic methods to assign labels to sequences from unknown organisms.

We propose a two-step method for taxonomy assignment where we use a rapid assignment method that can accurately assign labels to sequences that are well represented in the database, and then use more complex phylogenetic methods to classify only those sequences unclassified in the first step. In this work, we study whether and when a method can assign accurate taxonomic labels using a similarity search of a reference database. We employ BLAST because it is one of the most widely used similarity search methods [5]. However, it has been shown that the best BLAST hit may not always provide the correct taxonomic label [6]. Most taxonomic-assignment methods utilizing BLAST employ ad-hoc techniques such as recording the consensus label among the top five hits, or using a threshold based on E-value, percent identity, or bit-score [7–10]. Here we propose an alternative approach for detecting whether and when the top BLAST hits yield correct taxonomic labels. We model the problem of separating phylogenetically correct matches from matches to sequences from similar but phylogenetically more distant organisms as a problem of outlier detection among BLAST hits. Our preliminary results involving simulated and real metagenomic datasets demonstrate the potential of employing our method as a filtering step before using phylogenetic methods.

### Taxonomy assignment using BLAST

Several metagenomic analyses use BLAST to assign taxonomic labels to uncharacterized reads in a sample [7–9]. BLAST is a sequence similarity search tool, and it calculates an E-value and a bit-score to assess the quality of each match. An E-value represents the number of hits of equal or greater score expected to arise by chance. A bit-score can be understood as representing the size of the space one would need to search in order to find as strong a match by chance. However all 16S sequences are related, and therefore these scores, derived from a model of random sequences, do not provide simple information for separating sequences from different phylogenetic categories.

### BILD scores for multiple sequence alignment

Multiple sequence alignments employ scoring functions to assess the quality of columns of aligned letters. Such functions have included Sum-of-the-Pairs (SP) scores [11], entropy scores [12], tree scores [13, 14] and the recently developed Bayesian Integral Log-Odds (BILD) score [15, 16]. For local pairwise alignment, substitution scores are implicitly of log-odds form [17]. BILD scores extend the log-odds formalism to multiple sequence alignments. They may be used in numerous contexts such as the construction of hidden Markov model profiles, the automated selection of optimal motifs, and the selection of insertion and deletion locations, and they can inform the decision of whether to include a sequence in a multiple sequence alignment. BILD scores can also be used to classify related sequences into subclasses, as we describe below.

## Methods

Broadly, our approach constructs a multiple alignment from all the top hits obtained by comparing a query sequence to a database. We use BILD scores to determine whether the multiple alignment can be split into two groups that model the data better than does a single group. In essence, we find a subset of the sequences that are more closely related to one another and to the query than to the rest of the sequences in the multiple alignment. When there is no such subset i.e. when the single alignment models the data better, we leave the query unclassified and such a query sequence is then classified in the second step by a phylogenetic method.

### Processing query sequences

Let *S* be the set of sequences in the reference database, each with a taxonomic label, and *Q* be a set of uncharacterized reads (i.e. query sequences). We first align each sequence in *Q* to sequences in *S* using BLAST (*-max_target_seqs 100 -outfmt “6 qseqid sseqid pident length mismatch gapopen qstart qend sstart send evalue bitscore qseq sseq” -task megablast*). For each $q \in Q$, we construct the ordered set $S_q$ that contains the segments yielding the top 100 bit-scores, in decreasing order of their score. We discard all segments $l \in S_q$ where the BLAST alignment of *q* and *l* covers $\le 90\%$ of *q*. We use the BLAST-generated local alignments involving *q* to impose a multiple alignment ($M^{q}$) on the sequences in $q \cup S_q$. We ignore all locations in the local alignment where there is an insertion in the BLAST hit sequence.

### Scoring for multiple sequence alignments and cuts

**Fig. 1:**
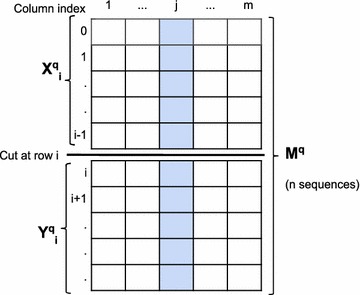
An example of how a cut divides an MSA into two disjoint groups

We base our score for a multiple alignment ($M^{q}$) on the Bayesian Integral Log-Odds (BILD) scores described in [15]. For each alignment column, we take the prior for the nucleotide probabilities to be a Dirichlet distribution with parameters $\mathbf {\alpha }$, and define $\alpha ^{*} = \sum _{k=1}^{4} \alpha _{k}$. (Here, we always use Jeffreys’ prior [18], for which all $\alpha _{k} = 0.5$, and $\alpha ^{*} = 2$). For the $j$th column $M^{q}_j$ of the alignment and ignoring null characters, the log-probability of observing its particular vector of $c^{*}_j$ nucleotides, with count vector $\mathbf {c_j}$, is then given by

$$\begin{aligned} L(M^{q}_j) = \log \Bigg [ \frac{\Gamma (\alpha ^{*})}{\Gamma (\alpha ^{*}+c_j^{*})}\prod _{k=1}^{4}\frac{\Gamma (\alpha _{k} + c_{jk})}{\Gamma (\alpha _{k})} \Bigg ]. \end{aligned}$$

Here, $\Gamma$ is a *gamma* function. As suggested in [15], the log-odds score for preferring a cut, at row *i*, of the column $M^{q}_j$ into the two sub-columns $X^{q}_{ji}$ and $Y^{q}_{ji}$, as illustrated in Fig. 1, is given by

1 $$\begin{aligned} V^{q}_{ji} = L(X^{q}_{ji}) + L(Y^{q}_{ji}) - L(M^{q}_j). \end{aligned}$$

Taking all columns into account, the log-odds score for preferring a cut at row *i* is simply formula 1 summed over all columns. However, we have found it useful to give greater weight to columns with greater diversity. Thus we adopt the score $V^{q}_i$ for a cut at row *i* given by the formula

$$\begin{aligned} V^{q}_i = \sum _{j=1}^{m} e^{a}_{j} V^{q}_{ji}, \end{aligned}$$

where $M^{q}$ has *m* columns, $e_{j} = - \sum _{k=1}^{4}{(c_{jk}/{c^{*}_j}) \log _4(c_{jk}/{c^{*}_j})}$ is the entropy (base 4) of column *j*, and *a* is an arbitrary positive parameter. Note that, using this formula, perfectly conserved columns have entropy 0 and thus weight 0, whereas columns with uniform nucleotide usage have entropy 1 and thus weight 1. We have found, by experimentation, that a useful value for the parameter *a* is 2.7.

### Outlier detection and taxonomy assignment

We are interested in finding the phylogenetically most closely related matches in the database to the query sequence *q*. We proceed by computing $V^{q}_{i}$ for cuts with increasing *i*, from $i=0$, and identify first $i'$ for which $V^{q}_{i'} \ge 0$, $V^{q}_{i'} > V^{q}_{(i'-1)}$, and $V^{q}_{i'} >V^{q}_{(i'+1)}$. In other words, we find the first peak among those scores that imply the data are better explained by a split alignment. (Scores below zero favor a single alignment). The first $i'-1$ sequences from $S_q$ we take as forming an outlier set $O_q = S_q[1:i'-1]$ for *q*. We extract the taxonomic labels of all sequences in $O_q$ and assign the lowest common ancestor (LCA; [10]) of these labels to *q*. In the case when scores favor a single alignment, we leave the query sequence unclassified. The unclassified query sequences then should be classified, in step two of a two-step process, using a phylogenetic method.

## Evaluation

### Datasets

We used the RDP 16S rRNA gene v16 dataset (RTS), which has taxonomy annotated for each of its 13,212 sequences [19], considering only the 12,320 sequences that had taxonomic labels for all six levels - Kingdom, Phylum, Class, Order, Family, and Genus. These sequences belong to 2,320 genera with, on average, 6 sequences per genus. To evaluate our outlier detection method, we compared taxonomic labels assigned to query sequences by our method to their true labels as given in RTS. First, we used V-Xtractor with default parameters to extract the V3, V4 and V3–V4 hypervariable regions of the sequences [20]. We then used these V3 (SIM-2), V4 (SIM-3), V3-V4 (SIM-4) and full (SIM-1) sequences as query datasets and RTS sequences as a reference database. We also used a real metagenomic dataset (Dataset-1) to study the effectiveness of our method in actual practice. Dataset-1 has 58,108 sequences from the V1–V2 hypervariable region.

### Leave-one-out validation

In the RTS simulated dataset, we know true taxonomic labels for all query sequences. For each taxonomic level, we compare the taxonomic labels assigned by our method to the true labels to find the number of queries that are correctly classified, misclassified or falsely unclassified. To identify correctly classified query sequences at each level, we compare, for all query sequences, the taxonomic labels assigned by our method to the true taxonomic label at that level. If the label assigned to a query by our method matches its true label, or if our method leaves the query sequence unassigned when there are no other sequences in the database with its particular label, we consider the query sequence as properly classified. For each taxonomic level, we consider misclassified those query sequences for which the assigned taxonomic label does not match the true label. We also consider falsely unclassified those sequences that were not assigned a taxonomic label at a particular level when the true label existed independently in the database.

**Fig. 2:**
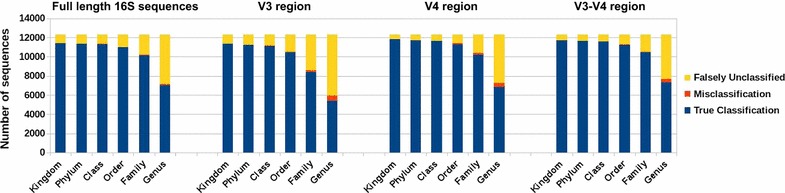
Leave-one-sequence-out validation of our outlier method using a simulated 16S rRNA dataset (RTS) for full-length, V3, V4, and V3–V4 regions

Figure 2 shows the number of correctly classified, misclassified and falsely unclassified sequences calculated by leave-one-out cross-validation, where we assign a taxonomic label to a query sequence (full or hypervariable region) after removing its associated sequence from the database. For all query datasets, our method rarely misclassified at all taxonomic levels, generally assigned correct labels at higher levels, but tended not to assign labels at lower levels. This may be because our method uses the LCA of taxonomic labels of outlier sequences. When there are closely related sequences in the database, our method chooses to be conservative by not assigning labels at lower taxonomic levels.

**Fig. 3:**
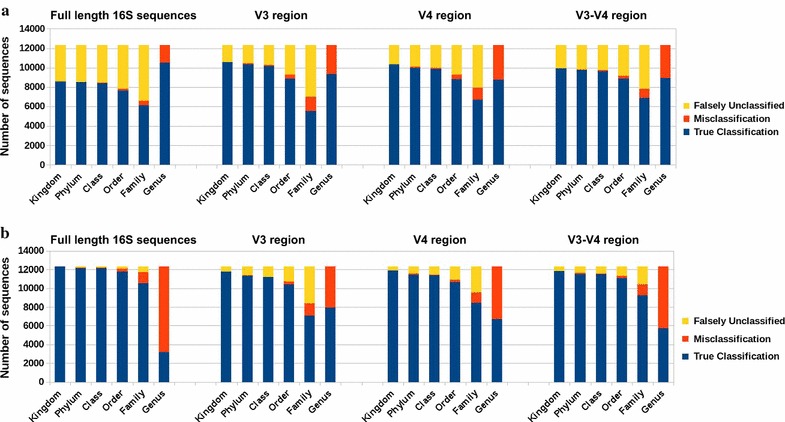
**a** Leave-one-genus-out validation of our outlier method using a simulated 16S rRNA dataset (RTS) for full-length, V3, V4, and V3–V4 regions** b** leave-one-genus-out validation of the RDP classifier on same 16S rRNA datasets

To study the effectiveness of our method in classifying sequences with taxonomy unrepresented in the database, we performed genus-level leave-one-out cross-validation. Specifically, for each query, we removed all sequences from the database belonging to the same genus, and assigned taxonomic labels with our method and the RDP classifier [21]. We ran the RDP classifier using the QIIME [22] pipeline with the default confidence threshold of $80\%$. We calculated the number of queries that were correctly classified, misclassified and falsely unclassified as explained above. Figure  3a and  b show results for our method and RDP respectively. Because the genus to which a query sequence belongs is never present in the database, any label assigned at genus level will result in a misclassification error, and no assignment will result in correct classification. We observed that for higher taxonomic levels (down to Order) RDP and our method have comparable misclassification rates. However, at the Family and Genus levels, our method has a lower misclassification rate. For all datasets, RDP misclassified more query sequences at the Genus level than did our method. This is primarily because RDP aggressively tries to classify as many sequences as it can, whereas our method prefers to classify only when it can do so accurately, leaving other sequences to be dealt with later by a phylogenetic method. This experiment shows that even when sequences from the same genus as the query are absent from the database, our method has high precision and makes few mistakes.

### Evaluation on a real 16S metagenomic dataset

**Fig. 4:**
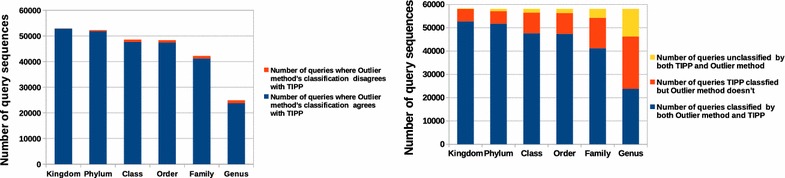
Evaluation of our outlier method using TIPP on a real metagenomic dataset.** a** Number of query sequences for which our method's classification agrees with TIPP's classification. **b** Number of query sequences classified by our method and TIPP versus unclassified both

**Fig. 5:**
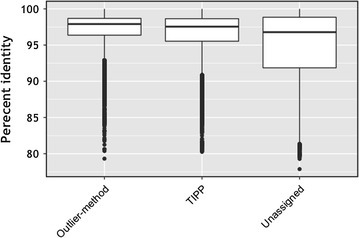
Box plot of percent identity of the best BLAST hit for all query sequences that were assigned label at genus level by our method and TIPP versus queries that remained unassigned by both methods

To study the effectiveness of our outlier detection method in a realistic setting, we tested it on a real metagenomic dataset. Since we do not know the true taxonomic label for all query sequences, we compared our results with those produced by TIPP [3], a phylogenetic-tree based taxonomic assignment method. We used the RDP 2014 16S reference database for both methods [19]. In this dataset, there were 58,108 query sequences for which our method assigned 41,256 sequences at the Family level or below. Figure 4a shows that our method has a high precision for all taxonomic levels. Also, Fig. 4b suggests that using our outlier method to make taxonomic assignments (at least down to the Family level) can significantly reduce the workload of a phylogenetic-tree based method like TIPP. A phylogenetic method can then search only in a subtree induced by database sequences in our outlier set as opposed to searching the whole tree for the best placement of the query sequence on the tree. About 11,000 sequences remained unclassified by both TIPP and our method, and we investigated whether the best BLAST hit’s percent identity correlates with the ability of these programs to make classifications; see Fig. 5. Unfortunately, there is no clear percent-identity cutoff one can employ to recognize sequences that will remain unassigned by both methods, although a large number of the unassigned sequences have low similarity to the nearest database sequence.

**Fig. 6:**
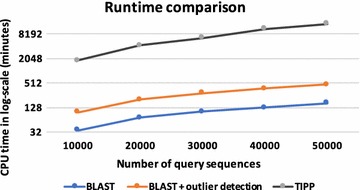
Runtime comparison of BLAST, BLAST+ outlier method and TIPP as a function of number of query sequences

We compared the running time of BLAST, BLAST+ outlier method, and TIPP on different input sizes. Figure 6 shows that both BLAST and our method have running time growing linearly with the number of query sequences whereas the running time of TIPP increases rapidly with the increase in the number of sequences. This shows that our method can be used as a quick and accurate pre-processing step before using a phylogenetic method.

### Distribution of outliers

**Fig. 7:**
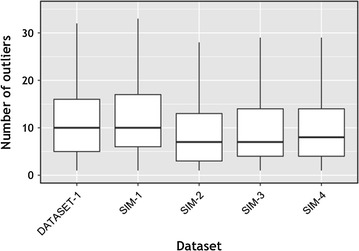
Box plot showing the variation in the number of outliers detected per query sequence in DATASET-1, SIM-1, SIM-2, SIM-3 and SIM-4.

**Fig. 8:**
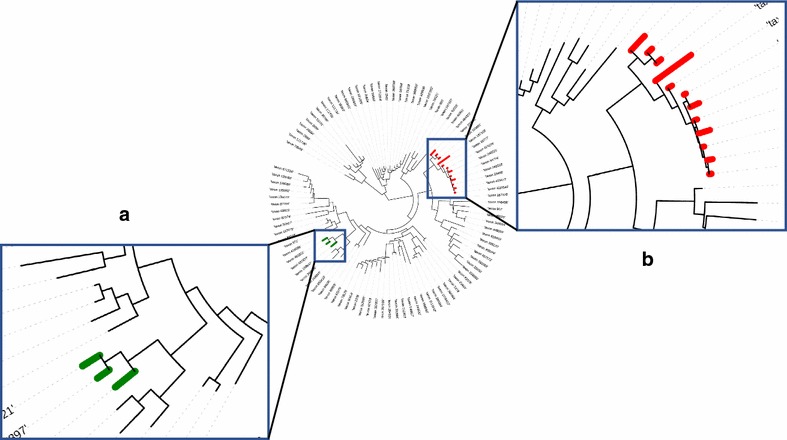
Phylogenetic tree showing outliers detected for two example query sequences. **a** sub-tree where the sequences identified as outliers are clustered closely to each other **b** sub-tree where the sequences identified as outliers cover a broader taxonomic range

Since prior approaches restrict the analysis to just a fixed number of top hits, we evaluated the number of outliers proposed by our method. As seen in Fig.   7, the number of outliers has large variance, so a single cutoff (say, the best or top five BLAST hits) will not identify all phylogenetically related matches from the database. In this case, we relied on data for which the true taxonomic label is not known. To validate whether the set of outliers detected by our method is reasonable, and to better understand the performance of our approach, we evaluated the placement of the outlier sequences within a phylogenetic tree of the database. For this, we used the phylogenetic tree for the RDP 2014 database that was bundled in the TIPP reference package, and used the Interactive Tree Of Life web tool to visualize outliers [23]. In general, we noticed that the outliers are grouped close to each other in the phylogenetic tree (see examples in Fig. 8), suggesting that our method produces reasonable results. This analysis also provided insights into the resolution level of the annotations produced by our method. When the outlier sequences cluster tightly within the phylogeny (Fig. 8a), a reliable classification can be made at a low taxonomic level. When the outliers are distributed across a broader section of the tree (Fig. 8b), the classification can only be made at a higher taxonomic levels.

### Effects of database and taxonomy

**Fig. 9:**
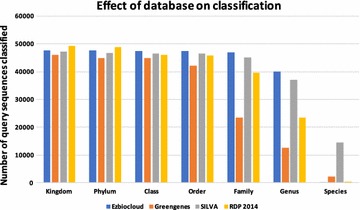
Number of query sequences classified by our method when using different databases in the BLAST search step

To understand the effect of the database on the final annotations provided by our method, we ran BLAST on four 16S rRNA gene databases—EzBiocloud, SILVA v.119, RDP 2014 and Greengenes on DATASET-1 [19, 24–26]. We used the Greengenes database from the QIIME package. It is known that these databases suffer from incorrect annotations. Mislabels can arise from the classification strategy used in curating the database or from errors in the current taxonomy, e.g. initial misidentification of species, or insufficient external sequence data for correctly arranging taxa [27]. Note also that these databases have different proportions of various taxa. Organisms that are well represented in a database will be classified more precisely whereas under-represented organisms will have labels assigned only at higher taxonomic levels. Thus, the differences in the number of sequences annotated by our method for different databases, as shown in Fig. 9, can be attributed primarily to the quality and the composition of the databases.

**Fig. 10:**
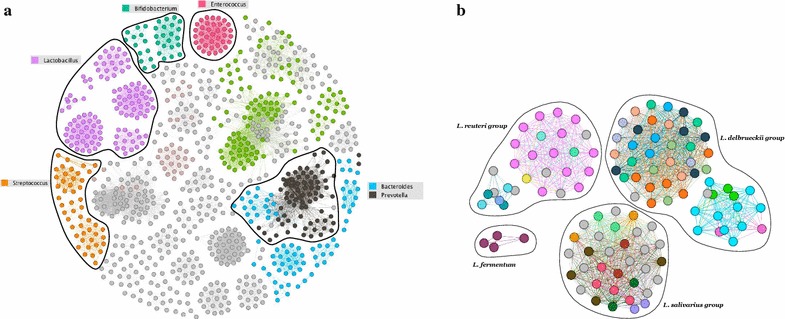
A graph where nodes are SILVA database sequences and edges between nodes are weighted by the number of query sequences from DATASET-1 for which the sequences of the two nodes are both present in the outlier set. We used the Gephi tool to visualize the graphs.** a** The connected components, when edges of weight less than 20 are removed, and where nodes are colored by the Genus label of the sequence.** b** The sub-graph of **a** showing only *Lactobacillus* species

A current taxonomy may not be fully resolved and our outliers can suggest refinements. For illustration, we constructed a weighted graph whose nodes are the sequences in the SILVA database, and with edges between two nodes weighted by the number of times the nodes co-occur in an outlier set. For this analysis, we again used $\sim 58$K query sequences from DATASET-1, keeping only the edges with weight at least 20. Figure 10a shows the connected components of the resulting graph colored and grouped by Genus. We used the Gephi tool to visualize these graphs [28]. Most of the components contain nodes of same Genus. For example, there is one component for *Enterococcus*, six for *Lactobacillus*, four for *Streptococcus*, etc. However, there are some Genera, such as *Bacteroides* and *Prevotella*, that have very similar regions in the V1–V2 segment of 16S rRNA gene. The query sequences matching these regions causes the edges in the graph and thus the two communities are not easily distinguished in our analysis. To analyze further the species distribution among these components, we examined the connected components for *Lactobacillus*, shown in Fig. 10b. We found that, within each component, all species belonged to a *Lactobacillus* group as defined by Felis et al. [29] and Salvetti et al. [30]. This shows that the outliers detected by our method can provide insights for resolving and refining a taxonomy. Alternatively, a user with information on deeper taxonomic levels can infer more detailed annotation for the species found in an outlier set than is provided by our method.

## Conclusion and discussion

We propose a two-step approach for taxonomic assignment, in which we gain as much information as we reliably can from BLAST output before using computationally expensive phylogenetic-tree based methods on sequences that are difficult to classify. In this paper, we developed an outlier detection method for taxonomy assignment using BLAST hits that separates phylogenetically correct matches from matches to sequences from similar but phylogenetically more distant organisms. This method can thus be used for step one of a two-step approach, to identify sequences that can be assigned accurate labels using just a BLAST search of a reference database.

Because all 16S rRNA sequences are related, statistics like BLAST’s E-value or bit-score do not provide ready information for separating sequences from different phylogenetic categories. Our experiments show also that there isn’t any single cutoff that can be used to select BLAST hits for correctly assigning taxonomic labels. We have experimented with finding outliers using bit-score distributions, but found they provided insufficient information to detect phylogenetically correct matches (data not shown). Our experiments also show that although the percent identity of its best BLAST hit is correlated to a sequence’s being assigned a taxonomic label, no particular percent-identity cutoff can separate those sequences that can be classified from those that cannot. This has motivated our development of a BILD-score based method to identify when the top BLAST hits will yield accurate taxonomic labels.

Because our method is used as a filtering step, we seek to accurately classify as many query sequences as possible while making few misclassifications. The sequences that we leave unclassified are then to be handled by a phylogenetic method. Our results on simulated and real 16S rRNA metagenomic datasets show that our method has high precision at all taxonomic levels, assigning correct labels at higher levels to a majority of sequences, and that it is computationally efficient compared to phylogenetic-tree based taxonomic assignment methods. This demonstrates the promise of a two-step taxonomic assignment approach, using our method as a filtering step.

In the future, we plan to study sequences that were classified correctly by phylogenetic methods but not by ours, to gain insight for possible improvements. We also plan to study the effectiveness of restricting phylogenetic-tree based methods to the subtree spanned by our method’s outliers. Finally, note that our method was developed for and tested on 16S rRNA data, and is not applicable as it stands to whole genome sequencing (WGS) datasets. However, the idea of using a two-step approach for taxonomy assignment in WGS datasets is an interesting avenue for research.
